# Activated T cell therapy targeting glioblastoma cancer stem cells

**DOI:** 10.1038/s41598-022-27184-w

**Published:** 2023-01-05

**Authors:** Ken Miyaguchi, Hongqiang Wang, Keith L. Black, Stephen L. Shiao, Rongfu Wang, John S. Yu

**Affiliations:** 1 Department of Neurosurgery, Cedars-Sinai Medical Center, 127 S. San Vicente Boulevard Suite A6600, Los Angeles, CA 90048 USA; 2 Department of Radiation Oncology, Cedars-Sinai Medical Center, 8700 Beverly Boulevard, Los Angeles, CA 90048 USA; 3 Department of Pediatrics, Children’s Hospital of Los Angeles, Department of Medicine, and Norris Comprehensive Cancer Center, The Keck School of Medicine at the University of Southern California, 1441 Eastlake Avenue, Los Angeles, CA 90033 USA

**Keywords:** Cancer immunotherapy, Cancer immunotherapy

## Abstract

Naïve T cells become effector T cells following stimulation by antigen-loaded dendritic cells (DCs) and sequential cytokine activation. We aimed to develop procedures to efficiently activate T cells with tumor-associated antigens (TAAs) to glioblastoma (GBM) stem cells. To remove antigen presentation outside of the immunosuppressive tumor milieu, three different glioma stem cell (GSC) specific antigen sources to load DCs were compared in their ability to stimulate lymphocytes. An activated T cell (ATC) protocol including cytokine activation and expansion in culture to target GSCs was generated and optimized for a planned phase I clinical trial. We compared three different antigen-loading methods on DCs to effectively activate T cells, which were GBM patient-derived GSC-lysate, acid-eluate of GSCs and synthetic peptides derived from proteins expressed in GSCs. DCs derived from HLA-A2 positive blood sample were loaded with TAAs. Autologous T cells were activated by co-culturing with loaded DCs. Efficiency and cytotoxicity of ATCs were evaluated by targeting TAA-pulsed DCs or T2 cells, GSCs, or autologous PHA-blasts. Characteristics of ATCs were evaluated by Flow Cytometry and ELISpot assay, which showed increased number of ATCs secreting IFN-γ targeting GSCs as compared with non-activated T cells and unloaded target cells. Neither GSC-lysate nor acid-eluate loading showed enhancement in response of ATCs but the synthetic peptide pool showed significantly increased IFN-γ secretion and increased cytotoxicity towards target cells. These results demonstrate that ATCs activated using a TAA synthetic peptide pool efficiently enhance cytotoxicity specifically to target cells including GSC.

## Introduction

Glioblastoma (GBM) is the most common and lethal brain tumor in the United States and accounted for 14.6% of all primary brain and other central nervous system (CNS) tumors in 2020^1^. Five-year survival rate after diagnosis is as low as 6.8%^1^. Different approaches have been studied to treat cancers including GBM. In particular, recent efforts in immunotherapy across multiple tumor types have seen tremendous success^2–5^. Given the well-known heterogeneity of GBM, immunotherapeutic strategies targeting multiple targets may hold the most promise for addressing this challenging disease.

Antigen specific cellular immune response is required to induce T cell (TC) activation by antigen presenting cells and proliferation in lymph nodes to initiate a T cell response against cancer. Dendritic cells (DCs) are professional antigen presenting cells with the greatest capacity to process and present antigen to T cells. Previously, we vaccinated GBM patients with a DC vaccine to induce a long-lived immune response in patients with GBM^6–8^. The antigen source for these trials included MHC-I acid-eluted peptides^6^, glioblastoma tumor lysate^7,8^, and specific epitopes targeting glioma stem cells (GSCs)^9,10^. The results showed that tumor antigen-loaded DCs can migrate to lymph nodes to induce antigen-specific systemic T cell responses that localize intracranially in glioblastoma^9^. A placebo-controlled randomized trial of those peptide loaded DCs demonstrated significant improvement in progression free survival in newly diagnosed GBM patients as compared to unloaded DC vaccinations^10^. The immune response was shown to target GSCs, which were derived from GBM patients. Despite these findings, the T cell responses generated by the vaccine remained limited in number and potency. This blunted response might be due to the lack of helper T lymphocyte (Th) support, which is critical to sustain a long-lived response^11–14^. In addition, cytokine support may be lacking in immunosuppressed cancer patients. To overcome these limitations, we sought to keep the potent antigen presentation of DCs supplemented with helper T cells and cytokines to support the effector response in a setting outside of the immunosuppressive mileu of the cancer patient. Immune cells that more directly attack cancer cells need to be highly strengthened in order to eliminate residual tumor and prevent recurrence after surgery, chemotherapy and radiotherapy. Naïve T cells from GBM patients can be activated ex vivo by autologous DCs loaded with cancer cell antigens and expanded to generate sufficient cytotoxic T lymphocytes (CTL), which can specifically target cancer cell antigens of GBM patients. The activation and expansion of T cells ex vivo enabled the increase in potency and numbers of cytotoxic and helper T cells for infusion.

To determine the best source of cancer cell antigens to target glioblastoma cancer stem cells, we compared the specific immunogenicity of antigens obtained from tumor lysate of GSCs, acid-eluted antigens from MHC-I epitopes washed from the surface of GSCs, and pool of specific MHC I and II peptides known to be expressed on GSCs. We sought to compare the generation of activated T cells (ATCs), which could target GSCs of GBM patients. Our goal was to develop a novel adoptive T cell therapy with the highest capacity to induce a specific T cell response against cancer stem cells in GBM, with a minimal risk of side effects.

## Results

### Characterization of T cells activated by glioma stem cell lysate

**Figure 1:**
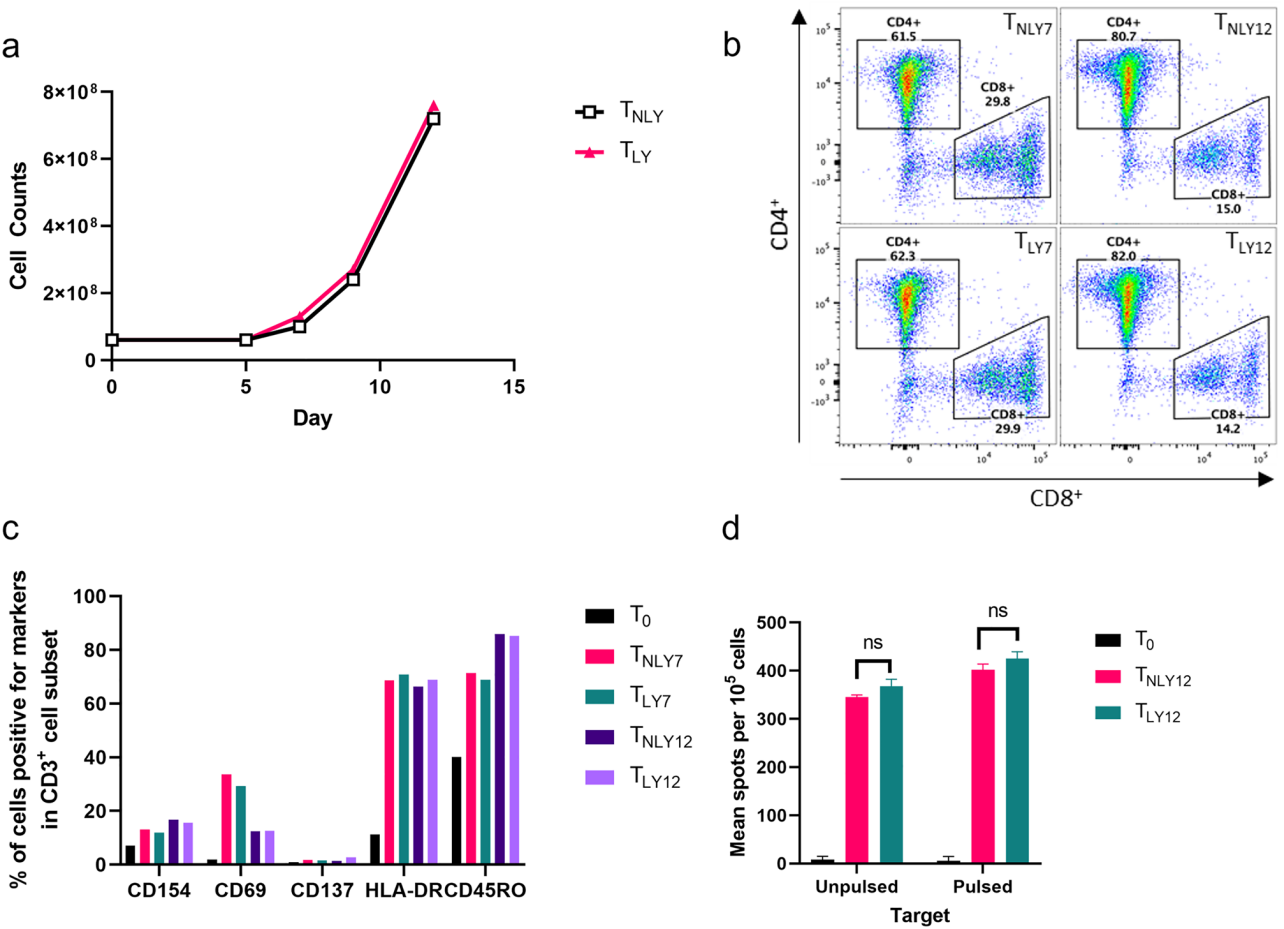
T cell activation with DCs loaded with GSC lysate. (**a**) Cell growth curve of activated T cells. 40 U/mL of IL-2 was added to the medium every other day until day 10. (**b**) Expression of CD4 and CD8 by CD3^+^ T cells at day 7 and 12. (**c**) Surface markers of T cells by flow cytometry. (**d**) ELISpot assay to measure IFN-γ secretion of T cells against DCs pulsed with or without GSC lysate. T cells without DCs were also measured as comparison. T_0_: naïve T cells, T_NLY12_: T cells co-cultured with non-lysate loaded DCs for 12 day, T_LY12_: T cells co-cultured with lysate-loaded DCs for 12 day. ns, no significant difference.

Autologous monocytes were differentiated into DCs with Granulocyte–Macrophage Colony Stimulating Factor (GM-CSF) and IL-4, then matured by lipopolysaccharide (LPS) and interferon-gamma (IFN-γ). GSC lysate (final concentration of 100 µg/mL) was loaded onto DCs to so that the mature DC are presenting peptides derived from GSC. Naïve T cells and DCs were then mixed at 10:1 ratio in a G-Rex 100 container for T cell activation and rapid proliferation for 12 days with culture medium containing IL-4, IL-7 and IL-2. Number of T cells expanded in the G-Rex container were counted at day 5, 7, 9 and 12 while IL-2 was added every other day to enhance the growth. Both T_NLY_ (T cells activated by unloaded DCs (DC-NLY)) and T_LY_ (T cells activated by GSC lysate-loaded DCs (DC-LY)) showed increases in cell density at day 12, which was 12 times great than the initial culture density (Fig. 1a). Co-cultured cells were sampled at day 7 and 12 for characterization analysis by flow cytometry to evaluate maturation of T cells. Sub-populations of CD3^+^ T cells were detected for CD4 and CD8. CD4^+^ T cells and CD8^+^ T cells were 82% and 14.2% of T_LY12_ cells, respectively while 80.7% of CD4^+^ and 15.0% of CD8^+^ T cells in T_NLY12_ were observed (Fig. 1b). There was no significant difference in CD4^+^:CD8^+^ ratio between T_LY12_ and T_NLY12_. More interestingly, over several days of incubation, CD4^+^:CD8^+^ ratio increased dramatically (62.3:29.9 on day 7 vs. 82:14.2 on day 12). Again, no difference was seen between T_NLY_ and T_LY_ groups. All surface markers including CD154, CD69, CD137, HLA-DR and CD45RO for T cell activation were up-regulated in all T cells compared to naïve T cells (T_0_) (Fig. 1c). To evaluate antigen-specific cytokine secretion as a result of activation, frequency of IFN-γ secreting T cells as effector cells against target cells, which were antigen-pulsed DCs or unpulsed DCs, was measured by ELISpot assay (Fig. 1d). Frequencies of IFN-γ secreting T cells were all up-regulated from T_0_. T cells at day 12 had more spot counts than ones at day 7 and especially against lysate-pulsed DCs compared to other target types (*p* < 0.01, data not shown). However, significant difference between T_LY_ and T_NLY_ was not detected in cytokine secretion against lysate-pulsed DCs (Fig. 1d).

### Characterization of T cells activated by acid-eluted peptides from GSCs

**Figure 2:**
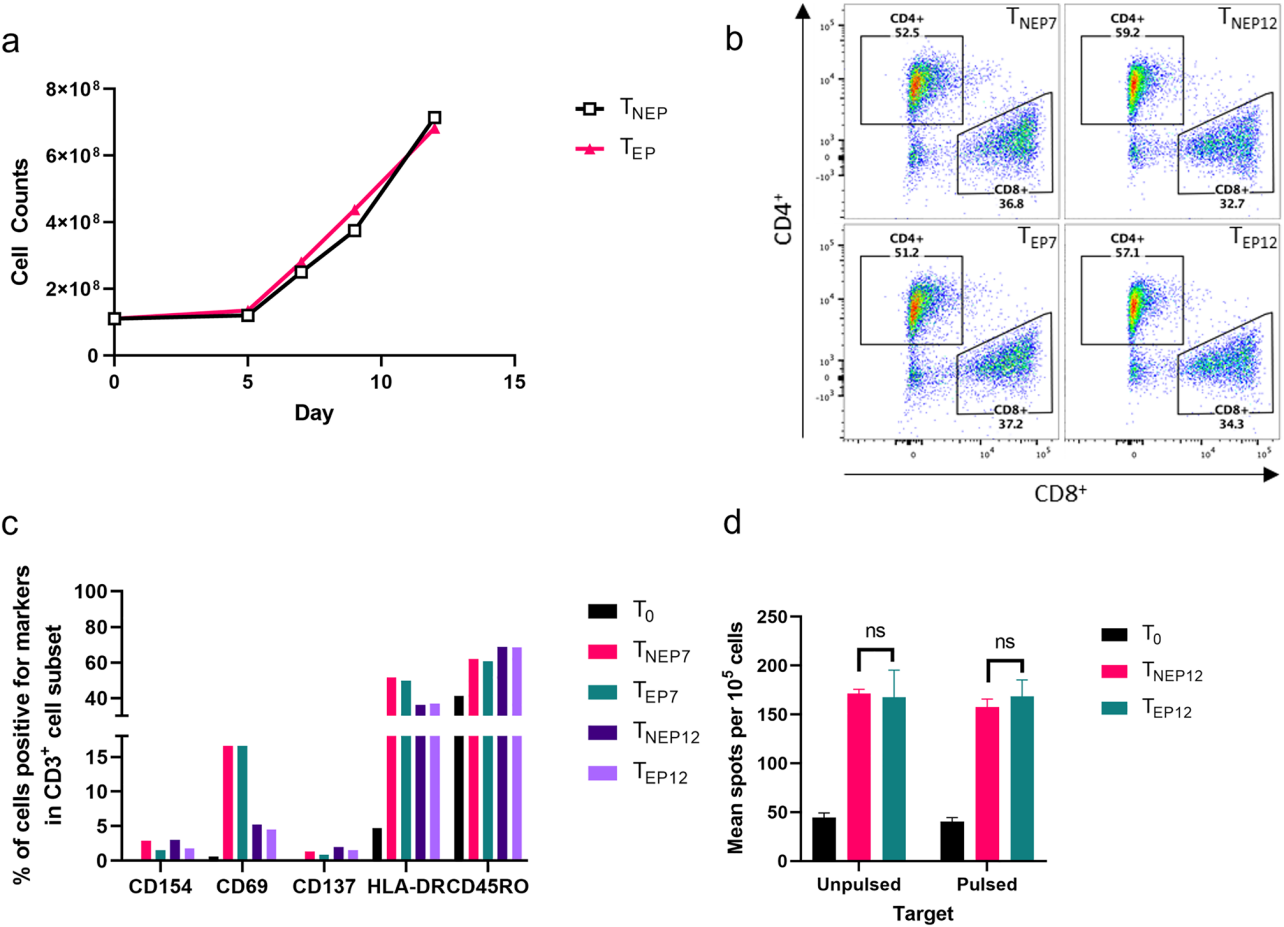
T cell activation with DCs loaded with GSC-eluted peptides. (**a**) Cell growth curve of activated T cells. 40 U/mL of IL-2 was added to the medium every other day until day 10. (**b**) Expression of CD4 and CD8 by CD3^+^ T cells at day 7 and 12. (**c**) Surface markers of T cells by flow cytometry. (**d**) ELISpot assay to measure IFN-γ secretion of T cells against DCs pulsed with or without GSC-eluted peptides. T cells without DCs were also measured as comparison. T_0_: naïve T cells, T_NEP12_: T cells co-cultured with non-peptide loaded DCs for 12 day, T_EP12_: T cells co-cultured with eluted peptides-loaded DCs for 12 day. ns, no significant difference.

Autologous monocytes were differentiated into DCs with GM-CSF and IL-4, then matured by LPS and IFN-γ. Acid-eluted peptides (100 ng/mL) were loaded to DCs following maturation. Naïve T cells and DCs were then mixed at 10:1 ratio in a G-Rex 100 container for T cell activation and rapid proliferation for 12 days with culture medium containing IL-4, IL-7 and IL-2. The number of T cells expanded in the G-Rex container were counted at day 5, 7, 9 and 12 while IL-2 was added every other day to enhance the growth. Both T_NEP_ (T cells activated by unloaded DCs (DC-NEP)) and T_EP_ (T cells activated by DCs loaded with eluted GSC peptides with acid (DC-EP)) showed increases in cell density at day 12, which was 6 times greater than the initial culture density (Fig. 2a). Co-cultured cells were sampled at day 7 and 12 for characterization analysis by flow cytometry to evaluate maturation of T cells. Sub-populations of CD3^+^ T cells were detected for CD4 and CD8. CD4^+^ T cells and CD8^+^ T cells were about 57.1% and 34.3% in T_EP12_ samples, respectively (Fig. 2b). On the other hand, CD4^+^ and CD8^+^ population of T_NEP12_ samples were 59.2% and 32.7%, respectively. There was no significant difference in CD4^+^:CD8^+^ ratio between T_EP12_ and T_NEP12_, or on day 7 versus day 12. All surface markers including CD154, CD69, CD137, HLA-DR and CD45RO for T cell activation were up-regulated in all T cells comparing to T_0_ (Fig. 2c). To evaluate antigen-specific cytokine production as a result of activation, frequency of IFN-γ secreting T cells as effector cells against antigen-pulsed DCs or unpulsed DCs was measured by ELISpot assay (Fig. 2d). Frequencies of IFN-γ secreting T cells were all up-regulated from T_0_ but significant difference among different types of T cells was not observed. T_NEP7_ cells always showed highest frequency.

### Generation of peptide pool as GBM specific antigens

Sequences of 9 CTL peptides and 9 helper T lymphocyte (Th) peptides were determined based on previous studies demonstrating expression on glioblastoma cells and on glioblastoma stem cells (Table 1) and potential cross-reactivities of ATCs activated with those synthetic peptides were analyzed. The Human Protein Atlas (https://www.proteinatlas.org/) was used to confirm the expression of each target protein in human normal tissues. RNA expression profiles in human normal tissues by NCBI Gene (https://www.ncbi.nlm.nih.gov/gene/) was used instead only if there was no data for the target protein in the Human Protein Atlas. According to the Human Protein Atlas, gp100, MAGE-A1, NY-ESO-1, and TRP-2 are uniquely expressed in normal skin, testis, testis and skin, respectively (data not shown). RNA expression of AIM2, EphA2, and IL-13Ra2 is relatively lower across any tissues except 1–2 tissue types such as lymph node, esophagus, and testis. HER2 protein is expressed in many types of normal tissue but it is known to be overexpressed in many types of cancer cells. We also performed homology searches by NCBI Protein BLAST (https://blast.ncbi.nlm.nih.gov/Blast.cgi) to investigate the cross-reactivity of our peptides. The search results showed that all epitopes were highly specific to each target protein except AIM2_Intron_ and gp100_209-217-2 M_ (data not shown). The peptide sequence of AIM2_Intron_ did not match to the original protein sequence because it was designed from the nonspliced transcript containing the retained intron, which is well recognized by T cells^15,16^. Sequence of gp100_209-217-2 M_ is modified from the original amino acid sequence of gp100_209-217_ [ITDQVPFSV]. We altered the peptide sequence [IMDQVPFSV] by changing the second amino acid from threonine (T) to methionine (M) as this was reported to increase the affinity for GM-CSF -associated HLA-A2.1, resulting in enhanced induction of T cells against native epitope^17–19^. These unique peptides did not show significant homology to other protein sequences.

**Table 1:** MHC class I and class II restricted epitopes.

Antigen	Sequence	Amino acids	HLA restriction	Reference
**MHC I**				
AIM2_Intron_	RSDSGQQARY	Nonspliced form	A1	Harada, (2001); Liu, (2004)
EphA2 _883–891_	TLADFDPRV	883–891	A2	Tatsumi, (2003); Pollack, (2014)
gp100 _209–217-2 M_	IMDQVPFSV	209–217	A2	Parkhurst, (1996); Akiyama, (2012)
HER2/neu _773–782_	VMAGVGSPYV	773–782	A2	Nguyen-Hoai, (2012); Phuphanich, (2013)
IL-13Rα2 _345–353_	WLPFGFILI	345–353	A2	Mintz, (2002); Okano, (2002)
MAGE-A1 _161–169_	EADPTGHSY	161–169	A1	Liu, (2004)
MAGE-A1 _278–286_	KVLEYVIKV	278–286	A2	Ottaviani, (2005); Shraibman, (2016)
NY-ESO-1 _157–165_	SLLMWITQC	157–165	A2	Wargo, (2009); Liu, (2018)
TRP-2 _180–188_	SVYDFFVWL	180–188	A2	Liu, (2003); Liu, (2005)
**MHC II**				
EphA2_663–667_	EAGIMGQFSHHNIIR	663–677	DR4	Tatsumi, (2003)
gp100_576–590_	SLAVVSTQLIMPGQE	576–590	DR7	Kobayashi, (2001)
HER2/neu_36–384_	KIFGSLAFLPESFDGDPA	369–384	DPB1*0401	Liu, (2004); Akiyama, (2012)
HER2/neu_688–703_	RRLLQETELVEPLTPS	688–703	DR4	Disis, (2002); Davis, (2003)
HER2/neu_883–899_	KVPIKWMALESILRRRF	883–899	DR1, DR4, DR52, DR53	Kobayashi, (2000)
HER2/neu_971–984_	ELVSEFSRMARDPQ	971–984	DR4	Knutson, (2001); Davis, (2003)
NY-ESO-1_119–143_	PGVLLKEFTVSGNILTIRLTAADHR	119–143	A68	Matsuzaki, (2008)
TRP-2_60–74_	QCTEVRADTRPWSGP	60–74	DRB1*0301	Paschen, (2005)
TRP-2_149–163_	KKRVHPDYVITTQHWL	149–163	DRB1*0301	Osen, (2010)

### Activation of autologous T cells by using GBM-specific-peptide pool

**Figure 3:**
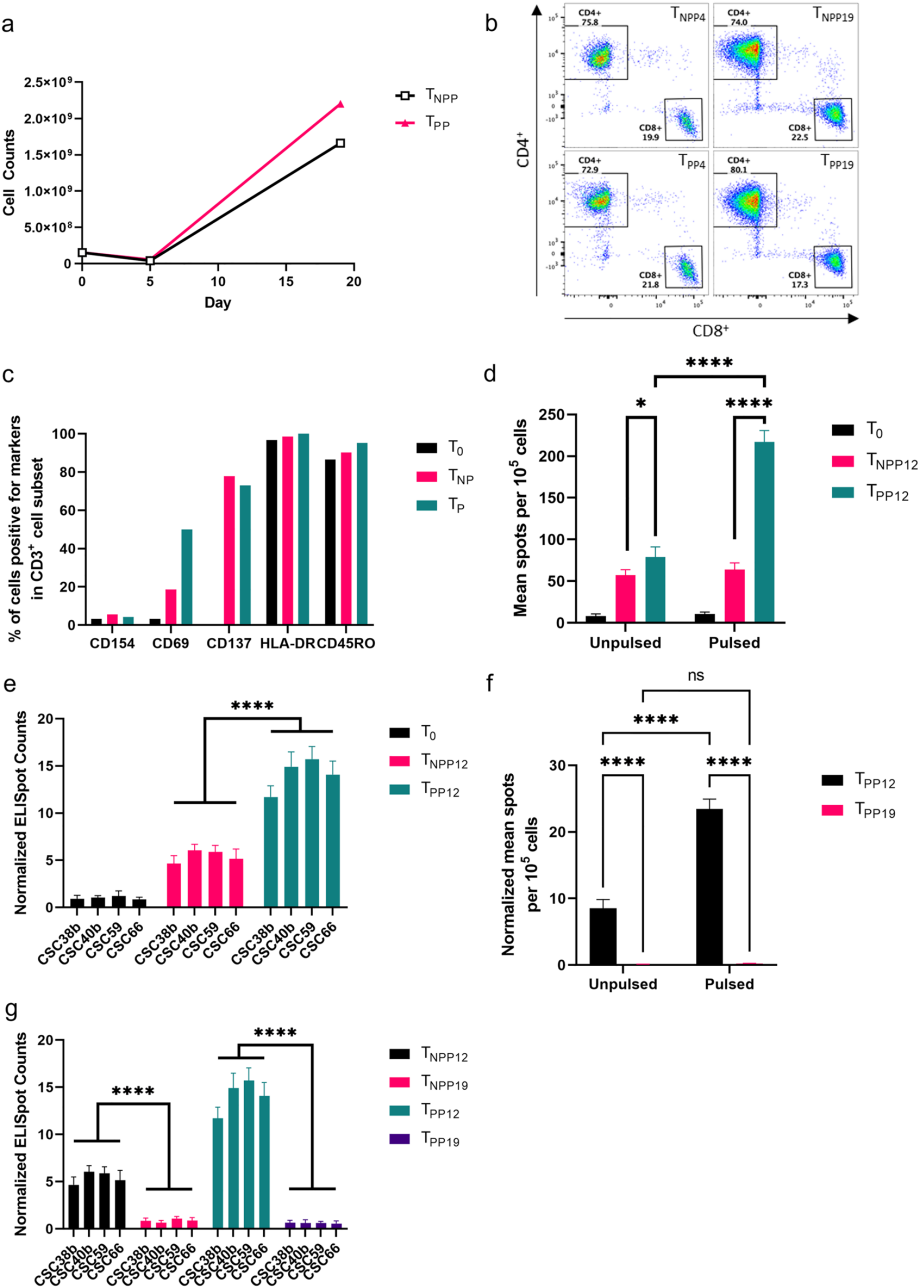
Autologous T cell activation by using GBM-specific peptide pool. (**a**) Cell growth curve of activated T cells. 40 U/mL of IL-2 was added to the medium every other day until day 19. (**b**) Expression of CD4 and CD8 by CD3^+^ T cells at day 4 and 19. (**c**) Analysis of T cell surface marker to assess activation of T cells at day 5. (**d**) ELISpot assay to measure IFN-γ secretion of T cells against GBM-specific peptides-pulsed T2 cells (T2-PP) in contrast to unpulsed T2 cells (T2-NPP). (**e**) IFN-γ secretion of T cells responding to institute-established GBM cancer stem cell lines (all HLA-A2 +) compared to naïve T cells (T_0_) and T cells activated by no-peptides-loaded DCs (T_NPP12_). (**f**) Comparison of IFN-γ secretion between 12 and 19 days of T cell activation by co-culturing ATCs with T2 cells pulsed with peptide pool. (**g**) Comparison of IFN-γ secretion between 12 and 19 days of T cell activation by co-culturing ATCs with four different GSC lines. *p < 0.05 and ****p < 0.0001. ns, no significant difference.

**Figure 4:**
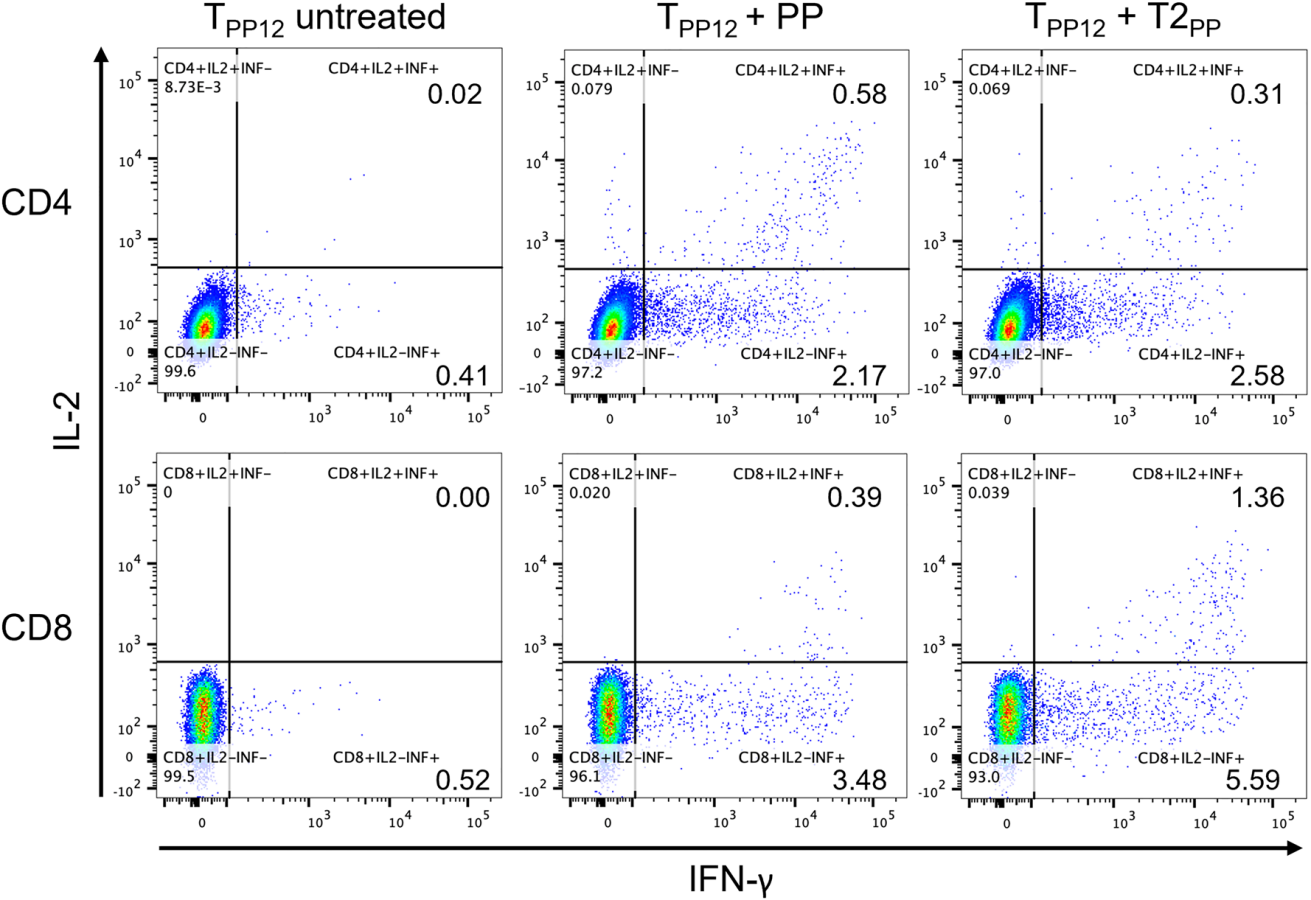
Intracellular cytokine staining. T cells were cultured under various conditions (as indicated) and stained for surface markers and cytokines (described in the materials and methods). The cells were first gated to exclude doublets or groups of cells, the live cells were selected by live/dead dye, followed by selection of CD3^+^ and CD4^+^ or CD8^+^. The cells could be further identified by cytokine expressions (the percentage of gated cells was indicated).

T cells were activated (T_PP_) by autologous monocyte-derived DCs, which were matured and loaded with synthetic peptide cocktail specific for GBM including 9 CTL epitopes (final concentration 20 µg/mL of each) and 9 Th epitopes (final concentration 20 µg/mL of each). As controls, T cells without incubation (T_0_) and T cells cocultured with DCs without peptide loading (T_NPP_) were also prepared. To assess the activation of T cells, T_PP_ and T_NPP_ were harvested at 5 days after co-culturing with DCs and analyzed for T cell surface markers by flow cytometry with T_0_. The number of T cells expanded in the G-Rex container were counted at day 5, and 19 while IL-2 was added every other day to enhance the growth. Both T_NPP_ (T cells activated by unloaded DCs (DC-NPP)) and T_PP_ (T cells activated by DCs loaded with GBM-specific synthetic peptide pool (DC-PP)) showed increases in cell density at day 19, which was 11 times larger for T_NPP_ and 14 times larger for T_PP_ than an initial culture density (Fig. 3a). Sub-populations of CD4^+^ and CD8^+^ in CD3^+^ T_PP19_ were 80.1% and 17.3% at day 19, respectively, and slightly increased for CD4^+^ (74%) and decreased for CD8^+^ (22.5%) in comparison with T_NPP19_ group (Fig. 3b). Compared with activation markers in T_0_, expression of CD69 and CD137 increased in T_NPP_ and T_PP_, furthermore, CD69 on T_PP_ group was much higher than T_NPP_, indicating T cells in T_NPP_ and T_PP_ were activated (Fig. 3c). There was no significant difference in expression of CD45RO and CD154 across all T cell types. T_NPP_ and T_PP_ were harvested at day 12 (T_NPP12_ and T_PP12_) and compared in ELISpot assay where T cells targeted two types of T2 cells (peptide pool-pulsed T2 (T2-PP) vs. unpulsed T2 (T2-NPP)) and patient-derived GSC lines CSC38b, CSC40b, CSC59 and CSC66, which are all HLA-A2 positive. Frequency of IFN-γ secreting T cells significantly increased when T_PP12_ was co-cultured with T2-PP and all four tested GSC lines (Figs. 3d and 4e, p < 0.0001). To determine the optimal culture time to obtain sufficient cytokine secretion of ATCs, ELISpot data for T cells co-cultured with DC-PP for 12 days (T_PP12_) and 19 days (T_PP19_) were compared against T2-PP and individual GSC line. Our results showed that more antigen specific IFN-γ secreting T cells were detected at day 12 than day 19 when T_PP12_ was co-cultured against antigen-loaded T2 cells or GSCs (Figs. 3f and 4g, p < 0.0001).

### Cytokine production of autologous T cells by intracellular cytokine staining

T cells from various groups: T_0_: naïve T cells at day 0, T_PP12_: T cells were expanded with peptide-loaded DCs for 12 days and T_NPP12_: T cells were expanded with unloaded DCs for 12 days, were in vitro cultured in three different conditions: medium alone (untreated), medium with peptide pool (PP), and medium with T2 cells pulsed with peptide pool (T2-PP). Our data showed that T_PP12_ had robust responses to T2-PP. Both CD4^+^ and CD8^+^ T cells from T_PP12_ group revealed a strong recall response to T2-PP with high IFN-γ secretion (Fig. 4). More interestingly, both CD4^+^ and CD8^+^ T cells presented multifunctionality: secreting both IFN-γ and IL-2. Although T_PP12_ under peptide pool stimulation also displayed high cytokine production from CD4^+^ and CD8^+^ T cells, as well as multifunctionality of T cells, in comparison with untreated group, but they are not as strong as T2-PP group. In addition, our data disclosed that T_0_ group as well as T cell group expended for 12 day without peptide pool stimulation (T_NPP12_) had minimal IFN-γ and IL-2 production, even with stimulations from T2-PP or PP alone (Table 2). In summary, our approach to generate tumor-antigen specific T cells demonstrated strong antigen-specific recall responses.

**Table 2:** Cytokine profile of various groups.

	T_0_			T_NPP12_			T_PP12_		
	Untreated	PP	T2-PP	Untreated	PP	T2-PP	Untreated	PP	T2-PP
% CD4^+^ IFN-γ	0.0032	0.0075	0.029	0.61	0.52	1.04	0.41	2.17	2.58
% CD4^+^ DP	0.0032	0.0075	0.012	0.013	0.013	0.042	0.02	0.58	0.31
% CD8^+^ IFN-γ	0.0052	0.009	0.39	0.53	0.45	1.41	0.52	3.48	5.59
% CD8^+^ DP	0.00	0.00	0.088	0.0056	0.0058	0.046	0.00	0.39	1.36
Total % IFN-γ	0.0084	0.016	0.42	1.14	0.97	2.45	0.93	5.65	8.17

*PP* peptide-pool, *DP* double positive for IFN-γ and IL-2.

% indicates percentage of gated cells.

### Cytotoxicity assay for glioma stem cells

**Figure 5:**
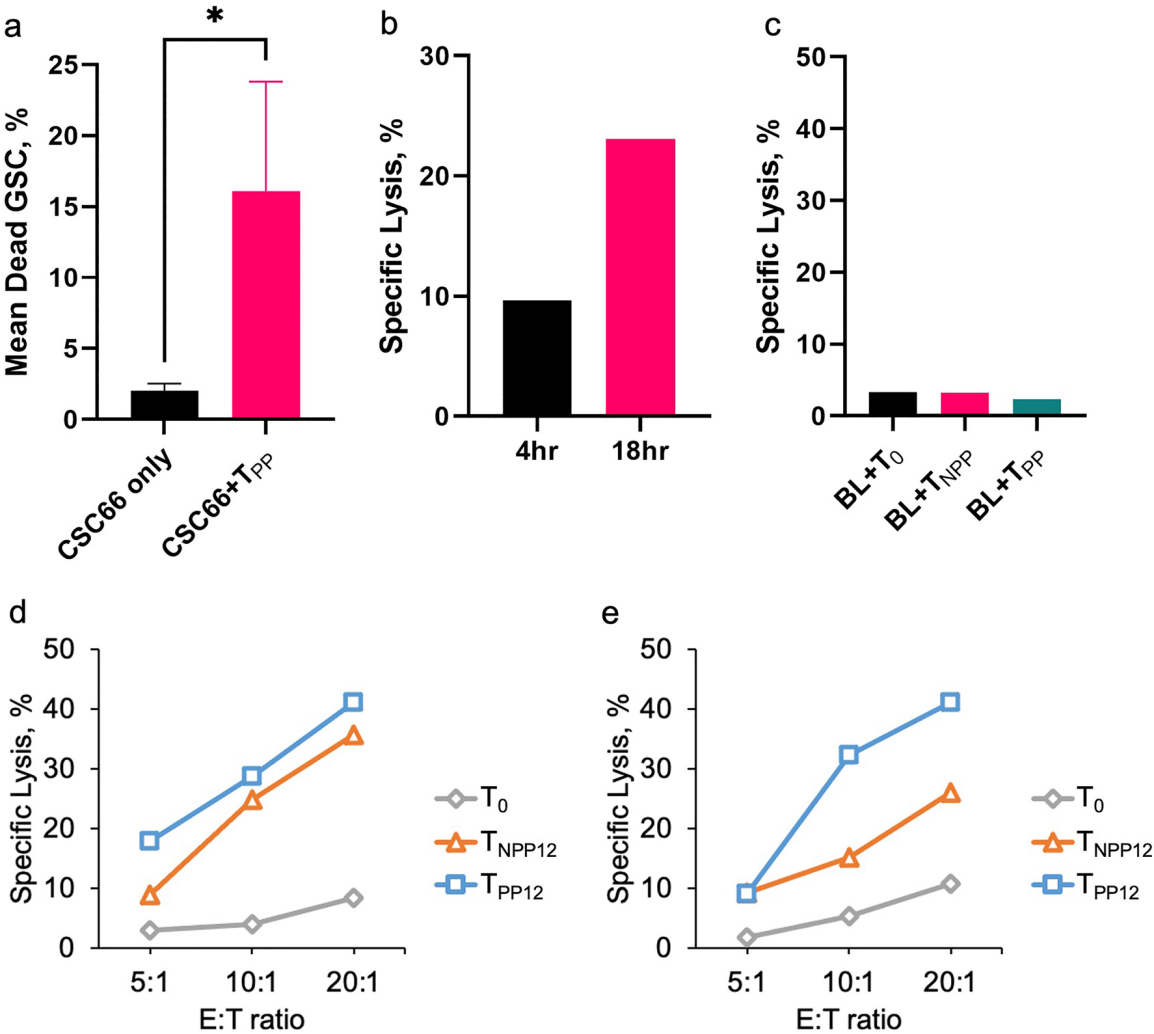
Cytotoxicity analyses with ATCs. (**a**) Population of dead CSC66 cells analyzed by flow cytometry. Dead GSCs were detected as double-positive for CellTrace Violet (CTV) dye and Fixable Viability eFluor 780 dye (FVD), where CTV stained only GSCs and FVD stained dead cells. The plot shows mean percentages of dead GSCs with or without co-culturing with T_PP_ (activated T cells by synthetic peptide-loaded DCs). (**b**) Comparison of specific lysis between short incubation (4 h) and long incubation (18 h). (**c**) In vitro assay for autoimmune effect of ATCs on autologous PHA-blast (BL). PHA-blasts were co-cultured with T_0_, T_NPP_ and T_PP_ at effector:target (E:T) ratio of 20:1. Population of dead PHA-blasts was also detected as spontaneous lysis by analyzing % parent CTV + /eFluor780 + population. % parent of PHA-blast without any T cells was used to calculate % specific lysis for T_0_, T_NPP_ and T_PP_, showing all less than 5% of dead PHA-blasts. (**d**) Percent specific lysis of floating U87 cells (detached from a culture plate and co-cultured as floating cells) lysed by T cells (T_0_, T_NPP12_ or T_PP12_) at different E:T ratio after co-culturing for 18 h. (**e**) Percent specific lysis of adherent U87 cells (kept attached on a culture plate and co-cultured as adherent cells) lysed by T cells (T_0_, T_NPP12_ or T_PP12_) at different E:T ratio after co-culturing for 18 h. Those percentages were calculated based on the population of dead U87 cells analyzed by flow cytometry. *p < 0.05.

Our data so far showed that among three various ways to generate antigen specific T cells, cells activated with GBM-specific peptide pool was the most robust generating cytokine. Here we further assessed the killing ability of these ATCs using CSC66 as target cells. As described in Methods, HLA-A2 positive GSC line, CSCC66 was stained with CTV dye, T cells with DC-PP (T_PP_) or DC-NPP (T_NPP_) were co-cultured at E:T ratio of 20:1. Single cell suspension was examined by flow cytometry for CTV dye. Mean percentage of dead CSC66 was 16.1% with effector T cells while mean spontaneous lysis of CSC66 was 2%, showing significant increase in killing target cells (Fig. 5a). Longer incubation time was reported to increase cytotoxicity of CTLs while standard 4-h incubation did not result in substantial cell killing^20^. Therefore, comparison between standard incubation (4 h) and overnight incubation (18 h) were performed to determine the optimal incubation time for cytotoxic killing assay on GSCs with ATCs. The longer incubation did not increase spontaneous cell death (data not shown) but percentage specific lysis increased more than double in 18-h incubation (Fig. 5b). There was no increased killing of HLA-A2 negative cells with any of our T cells (data not shown).

### Autoimmune responses by activated T cells

To assess the potential autoimmune side effect of activated T cells, autologous, phytohemagglutinin-L (PHA) stimulated lymphoblasts were generated from peripheral blood mononuclear cells (PBMCs) from the same donor and co-cultured with T cells at 20:1 (E:T)^21^. We compared percentage lysis of PHA-blasts among DC-PP activated T cells (T_PP_), non-activated T cells (T_NPP_) and spontaneous lysis of blasts measured by Flow cytometry (Fig. 5c). Plot shows that less than 5% of PHA-blasts were killed by any of T cells (T_0_, T_NPP_ or T_PP_). There was no significant effect of activated T cells on autologous immune cells.

### Cytotoxicity assay using the U87 GBM line

We also demonstrated an antigen specific dose-dependent killing pattern from an E:T ratio of 5:1 through 10:1 to 20:1 using the HLA-A2 positive U87 cell line^22^. This was consistent when using detached U87 cells (Fig. 5d) as well attached U87 cells (Fig. 5e). The cytotoxicity was over 40% under both conditions at the 20:1 E:T ratio.

## Discussion

In this study, we demonstrated the development of autologous activated T cell therapy using three different types of antigen stimulation to pulse DCs ex vivo in order to identify the optimal activation conditions to generate ATCs, which can improve the efficacy of cancer immunotherapy against GSCs. Cancer cell lysate has been used as one of the major antigen sources to load DCs against cancer cells and induce cytotoxicity of T cells^7,23–25^. However, in this study, cell lysate was not the most efficient stimulator to activate T cells specific to epitopes loaded on DCs as targets. T cell activation markers showed that T cells at day 7 were clearly activated when compared to naïve T cells (T_0_), which was also true for the cells at day 12. However, there was no significant difference between T cells co-cultured with DCs loaded with tumor lysate (T_LY7_) and T cells co-cultured with unloaded DCs (T_NLY7_) for 7 days (data not shown), or between T_LY12_ and T_NLY12_, which were expanded for 12 days. Using lysate from cancer stem cells may not be an ideal antigen source because cell lysate contains various types of proteins and debris besides relevant fragments of cancer specific target proteins as tumor antigen for DCs to present. Only limited number of DCs could be loaded with a relevant set of TAAs over a large amount of non-TAAs. In addition, the efficacy of lysate-based T cell activation might be limited if the cancer cells do not express sufficient numbers of antigens, suggesting that antigen source needs to be autologous not allogenic when cancer cell lysate is used to pulse DCs. Gonzalez et al.^25^, further mentioned pulsing DCs with tumor lysate can also induce tolerogenic transformation of DCs by providing immunoregulatory cytokines which may explain the poor activation observed with tumor lysates in our experiments^25^.

A similar explanation can be applied to the activation efficiency of T cells by acid-eluted GSC peptides. T cell activation markers showed that T cells at day 7 were clearly activated when compared to T_0_ cells, which was also true for the cells at day 12. All T cell groups showed more IFN-γ secreting cells than T_0_ cells, but there was no significant difference between DCs with or without the peptides. Although isolated acid-eluted peptides on the surface of GSCs contain less variety of peptides than whole GSC lysate, acid-eluted GSC peptides still may have limited antigen-specific activation of T cells against target cells.

Regardless of whether they are loaded with antigens or not, matured DCs induced activation of autologous naïve T cells (T_0_) in vitro. Compared to T_0_ cells, IFN-γ secretion by all ATCs encountering different stimulus was increased dramatically. T cells seemed to react strongly toward DCs loaded with antigen compared to “empty” DCs (no-antigen loaded). But no statistical significance was found in activation with DC-LY or DC-EP, suggesting that no matter how T cells are activated by DCs loaded with GSC-derived antigen (either GSC-lysate or acid-eluted peptides), the activation is not sufficient to produce tumor antigen-specific ATCs. However, in terms of difference between DC-LY and DC-EP, T cells activated by DC-LY showed much stronger responses as shown by the frequencies of IFN-γ secreting T cells following DC-LY than ones activated by DC-EP.

Delluc et al.^26^ demonstrated the comparison of immunogenic efficacy between two different acid elution methods, which were citrate–phosphate (CP) and trifluoroacetic acid (TFA) buffer, to choose the best elution method for DC-based vaccine therapy in acute myeloid leukemia (AML)^26^. Their study found that TFA-eluted peptide-loaded DC was more efficient to induce immune response specific to leukemia than CP-eluted peptide-loaded DC, suggesting that even the selection of the acid elution method to isolate cancer cell specific antigen peptides significantly affects to the immunogenic efficacy of generated ATCs. In this study we only used CP buffer to isolate dissociated peptides, as described in our previous Phase I trial^6^. Using TFA to elute peptides from the membrane surface of GSCs may improve the immunogenicity of ATCs. However, expectation of dramatic improvement is not high because cell lysate loaded DCs induce significantly higher number of IFN-γ secreting T cells than TFA-loaded DCs against an Epstein-Barr virus (EBV)-transformed B-lymphoblastoid cell line^27^.

To overcome the poor efficiency of both antigen sources (GSC lysate and acid-eluted peptides) in T cell activation, a pool of GBM-specific synthetic peptides was applied as the third stimulation method. Activation of T cells by DC-PP was confirmed by surface marker analysis and there were more IFN-γ secreting cells for antigen specific ATCs towards T2-PP as antigen presenting cells than any other conditions including ATCs with T2 cells without peptide pulsing. Although both T2 cells with or without synthetic peptide pool activated T cells, no significant difference of activation markers was shown between these groups. However, with regard to antigen specific cytokine secretion, there was a significant difference. This may suggest that T cell activation marker expression may not correlate with cytokine production function. More interestingly, our study suggests that T cell expansion with tumor peptide pool stimulation generates antigen-specific multifunctional T cells. Multifunctionality is the ability of T cells to perform several functions, such as the secretion of several cytokines, or other soluble lysis factors at the single cell level. It has been shown as an important parameter to predict the quality of T cell response and immunological control of tumor and infectious disease^28,29^. More studies are needed to understand the mechanisms to elicit multifunctional T cells in order to enhance tumor specific immunity.

We also investigated how longer incubation times for co-culturing T cells with DCs impacts T cell activation, which helped us to determine if longer culturing time can enhance cytotoxicity of ATCs. However, we found the opposite result with a dramatic decrease in the number of IFN-γ secreting T cells after an additional 7 days of culture. Longer incubation (12 days) induced higher frequency of IFN-γ secreting T cells than shorter incubation (3 days) (data not shown) but cytotoxicity lowered at day 19. These results indicate that cytotoxicity of ATCs becomes significantly high after 12 days of co-culture with DC-PP but excess culture diminishes the activation effect.

To assess the cytotoxicity of ATCs against GSCs in vitro, we performed non-radioactive cytotoxicity assay with of CellTrace Violet (CTV) dye instead of chromium 51 (^51^Cr) release assay^30,31^, which has been a traditional method for assessment of cytotoxic effect by CTLs in vitro^32,33^. Percentage of lysed GSCs dramatically increased by co-culturing with peptide-activated T cells. Autoimmune toxicity against normal cells should be evaluated when immunogenicity is induced because of the potential cytotoxic effect of ATCs against autologous healthy immune cells or normal tissues^34,35^. One of the major risks of T cell therapy is graft-versus-host (GVH) response when donor T cells are infused to the patient. However, this GVH adverse effect can be avoided if autologous T cells are activated and expanded ex vivo to treat cancer in the same patient. On the other hand, there still remains possible side effects^36^. Sequences of MHC I and MHC II peptides were carefully selected to enhance cytotoxicity against GSCs and minimize autoimmune reaction in normal cells, which has been reported when normal cells express the same peptides utilized as the antigen source for T cell activation^34,35^. T cells activated by this method kill less than 5% of autologous PHA-stimulated blast cells, suggesting that our T cells activated by DC-PP do not induce autoimmune toxicity by ATCs against autologous immune cells. Our results showed that the method to generate ATCs by a synthetic peptide pool was feasible and efficient to activate T cells generating antigen-specific immune responses without showing any autoimmune toxicity, supporting the notion that this method can be used as a safe way to propagate GSC-specific ATCs for immunotherapy for GBM.

In conclusion, a pool of cancer-specific synthetic peptides was found that exhibited as a promising source for T cell activation compared to other traditional sources of tumor antigen, which are tumor cell lysate or acid-eluted tumor cell peptides from a GSC line. We have established a potentially safe protocol to develop T cell therapy activated by synthetic peptide-loaded dendritic cells for GBM patients, showing enhanced capability of cytotoxicity against HLA type matched glioma stem cells in vitro without autoimmune risk. After infusion, these activated T cells should have the capacity to generate tumor antigen-specific and cytotoxic immune responses in vivo to facilitate GBM treatment. This method of T cell activation has been implemented into an investigational new drug cleared by the FDA.

## Materials and methods

### Patient-derived glioma stem cell lines

Patient-derived GSCs were established from tumors of GBM patients as previously described^37,38^ and cultured in serum-free neurobasal medium at Cedars-Sinai Medical Center. All GSCs were approved by the institutional review board of Cedars-Sinai Medical Center and informed consents were obtained from all patients. All methods were performed in accordance with the Cedars-Sinai Medical Center institutional review board and institutional biosafety committee guidelines and regulations.

### Monocytes and T cell preparation

HLA-A2 positive apheresis products were obtained from the Blood Donor Facility at the Cedars-Sinai Medical Center or purchased from HemaCare Corp. (Van Nuys, CA). Using the Elutra Cell Separation System (Terumo BCT), both monocytes and lymphocytes were isolated and red blood cells (RBCs) were removed. By staining the lymphocyte fraction with anti-CD3 antibody (BD Bioscience, Cat.564810) purification of T cells was assessed (T cells > 80%) and cells were frozen and stored in liquid nitrogen tank for later use. The monocyte fraction was stained with anti-CD14-PerCP (BD Bioscience, Cat.340585), anti-CD45-FITC (BD Bioscience, Cat.555482) and anti-CD66-PE (BD Bioscience, Cat.551480) antibodies. In order to generate DCs, more than 60% of the monocyte population needs to be CD14^+^/CD45^+^ while CD66^+^ cells are less than 10%.

### Cancer stem cell lysates

Cancer cell lysates of GSC was prepared by harvesting cultured GSCs and cell lysates generated by several freezing and thawing cycles. Briefly, cultured neurospheres were collected and then dissociated by pipetting. Cells were then washed with 1X DPBS and frozen down with freezing medium at − 80 °C. Then cells were thawed at room temperature until completely thawed, followed by freezing the cells at − 80 °C for more than 2 h or in liquid nitrogen for 30 min. The freeze–thaw cycles were repeated three more times for a total of 4 cycles. After the last thawing step, the resulting suspension was centrifuged at 2000 rpm at 4 °C for 10 min. The supernatant was collected, filtered through 0.2 µm syringe filter and stored at − 80 °C until use.

### Acid elution of peptides

MHC-I associated peptides were isolated from the cell surface proteins by eluting GSC lysate with acid as previously described^6,39^. Cultured GSC spheres were collected and dissociated. After several washings with HBSS buffer, about 10^8^ cells were resuspended in 10 mL of citrate–phosphate buffer (pH 3.2) to dissociate peptides from surface MHC-I, gently homogenized until cell disruption and centrifuged at 1000 rpm for 5 min. The supernatant was collected and then concentrated by passing through Sep-Pak C18 cartridge (Waters Corp.), aliquoted and stored at − 80 °C.

### Synthetic peptides

Eighteen human GSC-specific peptides including 9 MHC-I peptides (CTL peptides) and 9 MHC-II peptides (Th peptides) were selected based on amino acid sequences previously reported (Table 1)^9,13,15–17,19,40–57^. All peptides were synthesized by PolyPeptide Group (Torrance, CA), diluted in dimethyl sulfoxide (DMSO) at 1 mg/mL and stored at − 20 °C until use. All eighteen peptides were pooled as antigen cocktail to stimulate DCs to later activate naïve T cells to become GSC-specific CTLs and Ths.

### Generation of dendritic cells

Monocytes were cultured in MACS GMP Cell Differentiation Bags (Miltenyi Biotec, Germany) containing DC culture medium (DC medium, 560 U/mL GM-CSF, 35 µg/mL IL-4, 1% Gentamycin) at 37 °C, 5% CO_2_ for 4 days. For DC maturation, 2000 U/mL of interferon-gamma (IFN-γ) and 60 U/mL of lipopolysaccharide (LPS) were added into the culture bag and the cells were kept culturing for another 24 h (GSC lysate was added at this step). Antigen source (for synthetic peptide cocktail, each peptide with final concentration of 20 µg/mL) was added into the culture bag on day 5 and the cells were incubated at 37 °C, 5% CO_2_ overnight (16–20 h). DCs were harvested in HBSS and their phenotype was evaluated by the flow cytometry (CD11c^+^/CD83^+^ population > 60%).

### ATC generation (Loading DCs with cancer cell specific antigens)

**Figure 6:**
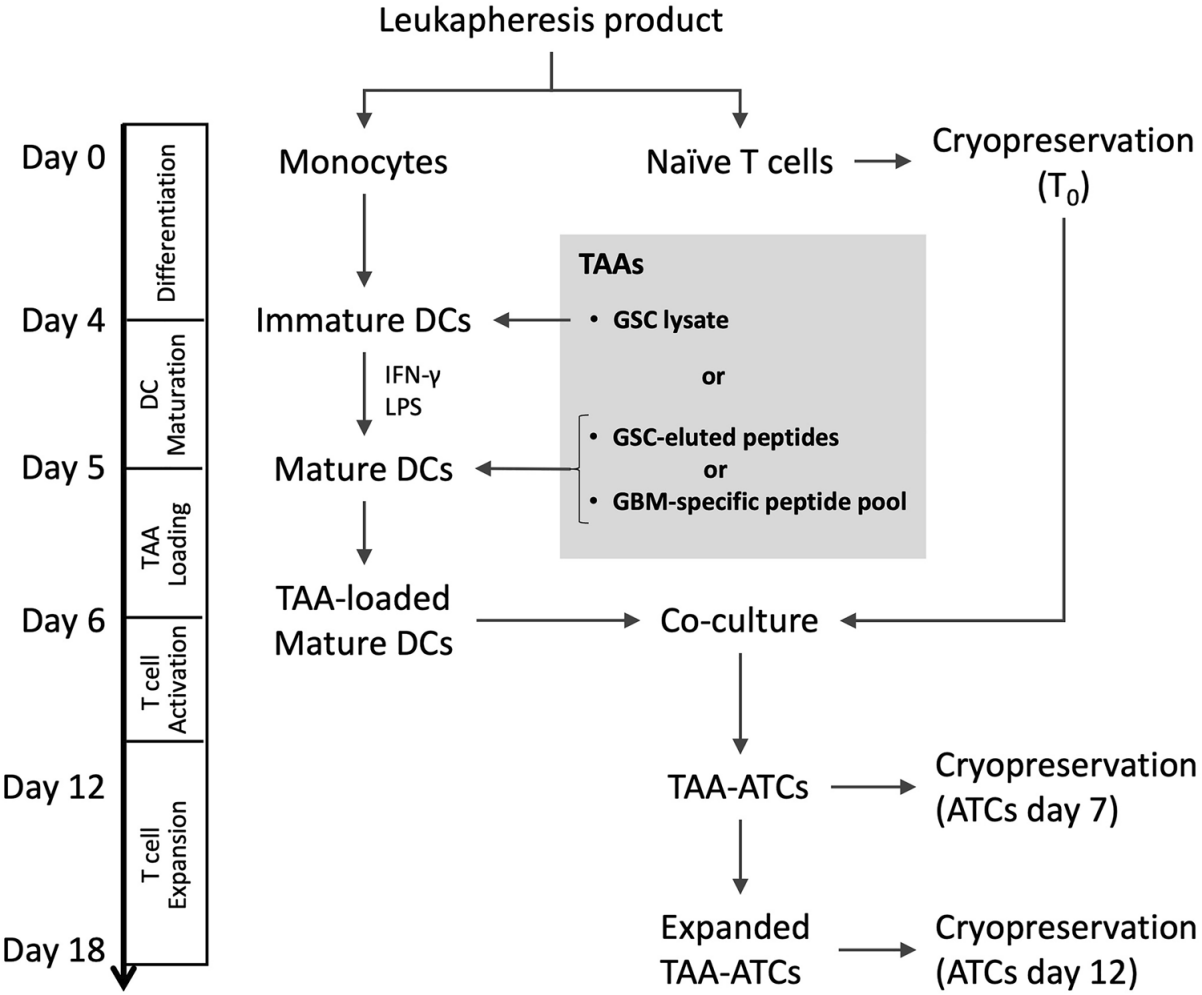
Workflow of producing tumor-associated antigens (TAA)-activated T cells. There are four main steps after isolation of monocytes and naïve T cells from donor’s leukapheresis product by elutriation. Isolated naïve T cells were cryopreserved for later use. Immature DCs were differentiated from monocytes and loaded with TAA (GSC lysate, GSC-eluted peptides or GBM-specific peptide pool) at day 4. TAA-loaded mature DCs were co-cultured with thawed naïve T cells to activate T cells specific for TAA (TAA-ATCs), followed by expansion of TAA-ATCs to obtain enough amount. TAA-unspecific ATCs were also generated by co-culturing naïve T cells with TAA-unloaded DCs. ATCs were cryopreserved at day 7 and 12 for comparison.

On the same day as matured DCs were harvested, frozen naïve T cells were thawed and allowed to recover in R10 medium (RPMI1640, 10% FBS) for 2 h (Fig. 6). Then 2 × 10^8^ cells of naïve T cells (T_0_) were co-cultured with 2 × 10^7^ cells of peptide-loaded DCs in the TC:DC ratio of 10:1) in G-Rex100 container (WilsonWolf Manufacturing Corp., P/N 80,500) with 200 mL T cell culture medium (TCM: RPMI1640, 45% Click’s medium, 10% FBS, 10 ng/mL IL-7 (CellGenix, Cat.1010), 400 U/mL (35 µg/mL) IL-4 (CellGenix, Cat.1003), 1% Gentamycin) at 37 °C, 5% CO_2_ for rapid growth and expansion. After 2 days, 40 U/mL of IL-2 was added into the culture medium and IL-2 was replenished every other day until day 10. IL-4 and IL-7 were replenished with fresh TCM into the culture at day 5 and 10. The cells were harvested at day 12 (T_NPP12_ and T_PP12_) according to the manufacturer’s instructions and surface markers were analyzed to confirm success of activation and ELISpot assay performed on final product to measure the IFN-γ secretion towards specific tumor antigen or cancer stem cell lines from which the antigen source was derived^12^.

### Characterization of activated T cells and quality control

ATCs were analyzed by flow cytometry for the surface markers including CD69, HLA-DR, CD45RO, CD154 and CD137 at day 5 and 12 in addition to CD3, CD4 and CD8. Early activation markers were noted to be highest on day 5 and maximal T cell growth was noted on day 12. Additionally, sterility testing for endotoxin, mycoplasma, gram stain and environmental culture was performed to ensure quality of the cell products for future clinical trials. Antibodies (BD Bioscience) used were anti-human CD3 (Cat. 564,810), CD4 (Cat. 555,346), CD8 (Cat. 560,662), CD69 (Cat. 562,883), HLA-DR (Cat. 564,231), CD45RO (Cat: 561,136), CD154 (Cat. 557,299) and CD137 (Cat. 561,702). Growth of ATCs was also measured along with the activation marker analysis. Maximal growth of T cells was noted to be at day 12 (data not shown).

### Interferon-gamma (IFN-γ) ELISpot assay

To quantify IFN-γ secreting cells as a result of the recognition of antigens on the surface of target cells by activated T cells, autologous DC or T2 cell, which is HLA-A2 positive lymphoblast cell line, was used as antigen presenting target cell. Antigen-pulsed DCs were co-cultured with T cells activated with either tumor-lysate or acid-eluted peptides-loaded DCs. T cells activated with synthetic peptide loaded DCs were co-cultured with antigen-pulsed T2 cells (Bossi, Oncoimmunology, 2013). For preparing T2 cell targeting assay, T2 cells were cultured in IMDM medium (20% FBS, antibiotics) and antigen peptides cocktail (final concentration of 5 µg/mL for each peptide) was pulsed for 2 h. Cryopreserved T cells (pre- and post-activation) were allowed to recover in R10 medium (RPMI1640, 10% FBS, 1% Gentamycin) for 2 h. For GSC targeting assay, four GSC lines were harvested and resuspended in ELISpot medium (RPMI1640, 10% FBS, 1% Gentamycin, 20 ng/mL IL-7). These cells were transferred to ELISpot plate (MabTech, Cat. 3420-4HPT-2) at TC to T2 ratio of 2:1 and TC to GSC ratio of 1:1.5 following the manufacturer’s protocol. Each well contained 1 × 10^5^ T cells with 200 µL medium. Additional wells for negative (medium only) and positive (anti-CD3 antibody) controls were also included. The plate was incubated at 37 °C, 5% CO_2_ overnight. After centrifuging at 1300 rpm for 3 min, supernatant was discarded, each well was stained according to the manufacturer’s protocol (Mabtech, Cincinnati, OH). Then the plate was sent to ZellNet Consulting (Fort Lee, NJ) for plate reading and quantification using ELISpot reading system (Carl Zeiss, Thornwood, NY). Pre- and post-activated T cells were analyzed for interferon γ-secreting T cells.

### Intracellular cytokine staining

T cells from various experiment groups: T_0_, T_NPP12_ and T_PP12_ were cultured in complete RPMI medium, or with T2 cell (pulse with synthetic peptide pool for 2 h on rotator), or with peptide pool for 4 h, followed by adding GolgiPlug (Biolegend, San Diego, CA). To exclude dead cells, samples were stained with Live/Dead dye (Biolegend, San Diego, CA), followed by surface staining (fluorochrome-conjugated antibodies: anti-CD3, anti-CD4, and anti-CD8, Biolegend, San Diego, CA) for 30 min on ice. Cells were then fixed with Fix & Perm Cell Fixation & Permeabilization Kit (ThermoFisher) according to the instruction, stained with fluorochrome-conjugated anti-IFN-γ and anti-IL-2 (Biolegend, San Diego, CA) for 30 min on ice, washed and resuspended in staining buffer with 1% PFA, followed by flow cytometry analysis by BD LSR Fortessa (Beckman Dickinson, Franklin Lakes, NJ.). Flow cytometry data was analyzed by FlowJo (Three Star, Ashland, OR).

### Generation of PHA-blasts

Autologous PHA-blasts were generated by stimulating PBMC with 5 µg/mL Phytohemagglutinin-L (PHA-L, Sigma, L2769), followed by culturing in RPMI1640 medium containing 10% FBS and 100 U/mL IL-2 (Gibco, PHC0027) in 24-well plate for about one week^12,14,21^. At day 3 or 4, cell growth and formation of blasts were assessed by microscopy. After 7 days of culture, PHA-blasts were harvested and frozen down in freezing medium (culture medium + 10% DMSO).

### Cytotoxicity assay

HLA-A2 positive GSC line, CSC66 or HLA-A2 negative GSC line, CSC55 were cultured in serum-free neural basal medium and harvested to use in a cytotoxic assay to evaluate efficiency of cell lysis by activated T cells. U87 cell line, which is HLA-A2 positive^22^, was also used as a target cell in a cytotoxicity assay. Collected cancer cells were stained as target cells with 2.5 μM of CellTrace Violet (CTV) dye (ThermoFisher Scientific, C34557) for flow cytometry analysis. T cells with peptide-loaded DCs (T_PP_) or non-peptide loaded DCs (T_NPP_) were cultured for at least 2 h prior to the assay. Those T cells were co-cultured with stained cancer cells in the effector to target cells (E:T) ratio of 5:1, 10:1 and 20:1 in 6-well plate at 37 °C for 4 h or 18 h. U87 cells were split into two groups where one group was trypsinized prior to co-culture (detached), and another group was co-cultured as adherent cells (attached). Cancer cells without T cells were also incubated in the same manner to detect basal cell death for the calculation of specific cell lysis by T cells. After the incubation, cells were stained with eBioscience Fixable Viability Dye (FVD) eFluor 780 (Thermo Fisher Scientific, 65–0865) and fixed with 4% formaldehyde, followed by filtering through 70 μm and 40 μm cell strainers. Single cell suspension was transferred to FACS tube and stored at 4 °C protected from light until use in flow cytometry analysis. Percentages of dead cancer cells (CTV-positive/FVD-positive) were measured by BD LSRFortessa flow cytometry. The percentage of specific lysis was calculated as below^58^.

1 $$ \% Specific\; Lysis = \frac{{\left( {\% Sample\; Lysis - \% Basal\; Lysis} \right)}}{{\left( {100\% - \% Basal \;Lysis} \right)}} \times 100\% $$

### Testing for autoimmune responses

Thawed autologous PHA-blasts were cultured in RPMI1640 medium containing 10% FBS and 100 U/mL IL-2 in 24-well plate for 1 to 3 days prior to use in a cytotoxic assay to evaluate autoimmune effect by activated autologous T cells. Collected PHA-blasts were stained as target cells with 2.5 μM of CellTrace Violet (CTV) dye (ThermoFisher Scientific, C34557) for flow cytometry analysis. T cells with peptide-loaded DCs or non-peptide loaded DCs were cultured for at least 2 h prior to the assay. Those T cells were co-cultured with stained PHA-blasts in the effector to target cells (E:T) ratio of 20:1 in 6-well plate at 37 °C for 4 h^21^. PHA-blasts without T cells were also incubated in the same manner to detect basal cell death for the calculation of specific cell lysis by T cells. After the incubation, cells were stained with eBioscience Fixable Viability Dye (FVD) eFluor 780 (Thermo Fisher Scientific, 65–0865) and fixed with 4% formaldehyde, followed by filtering through 70 μm and 40 μm cell strainers. Single cell suspension was transferred to FACS tube and stored at 4 °C protected from light until use in flow cytometry analysis. Percentages of dead PHA-blast cells (CTV-positive/FVD-positive) were measured by BD LSRFortessa flow cytometry. The percentage of specific lysis was calculated by the same equation used for cytotoxicity assay^58^.

### Comparison of incubation times for cytotoxicity assay

Frozen T_PP_ cells were thawed and cultured for at least 2 h prior to the cytotoxicity assay described above. T cells were co-cultured with stained GSCs in the effector to target cells (E:T) ratio of 20:1 in 6-well plate at 37 °C for 4 h (short incubation) and 18 h (long incubation). GSCs without T cells were also incubated in the same manner to detect basal cell death for the calculation of specific cell lysis by T cells. After the incubation, cells were stained and analyzed by flow cytometry in the same manner as described above. % specific lysis between short incubation and long incubation was compared.

### Statistical analysis

ELISpot data were analyzed with two-way ANOVA followed by Tukey’s test to adjust for multiple group comparisons. Cytotoxicity data were analyzed with Student’s *t*-test. Residuals were inspected to confirm homoscedasticity and normality. Data were considered significant where *p* < 0.05. Data are presented as means + / − standard deviation (SD). Analysis performed with GraphPad software (v9.1). Each experiment was performed with at least triplicate replicates.

## Data Availability

All data generated in this study are available from the corresponding author on reasonable request.
